# Effects of intravenous infusion of lidocaine and dexmedetomidine on inhibiting cough during the tracheal extubation period after thyroid surgery

**DOI:** 10.1186/s12871-019-0739-1

**Published:** 2019-05-04

**Authors:** Shenghong Hu, Yuanhai Li, Shengbin Wang, Siqi Xu, Xia Ju, Li Ma

**Affiliations:** 1 Department of Anesthesiology, The First Affiliated Hospital, Anhui Medical University, Hefei, 230022 China; 2 Department of Anesthesiology, The Anqing Affiliated Hospital, Anhui Medical University, Anqing, 246003 China; 3 Department of Thyroid and Breast Surgery, The Anqing Affiliated Hospital, Anhui Medical University, Anqing, 246003 China

**Keywords:** Lidocaine, Dexmedetomidine, Cough, Thyroid surgery

## Abstract

**Background:**

Intravenous lidocaine and dexmedetomidine treatments have been proposed as methods for inhibiting cough. We compared the efficacy of intravenous lidocaine and dexmedetomidine treatments on inhibiting cough during the tracheal extubation period after thyroid surgery.

**Methods:**

One hundred eighty patients undergoing thyroid surgeries were randomly allocated to the LIDO group (received lidocaine 1.5 mg/kg loading, 1.5 mg/kg/h infusion), the DEX group (received dexmedetomidine 0.5 μg/kg loading, 0.4 μg/kg/h infusion) and the CON group (received saline), with 60 cases in each group. The primary outcomes of cough were recorded. Secondary outcomes included hemodynamic variables, awareness time, volume of drainage, the postoperative visual analogue scale and adverse effects were recorded.

**Results:**

The incidence of cough were significantly lower in the LIDO group (28.3%) and the DEX group (31.7%) than that in the CON group (66.7%) (*P* = 0.000). Additionally, both moderate and severe cough were significantly lower in the LIDO group (13.3%) and the DEX group (13.4%) than these in the CON group (43.4%) (*P* < 0.05). Compared with the two treatment groups, both mean arterial blood pressure and heart rate were significantly increased in the CON group during tracheal extubation (*P* < 0.05). Compared with the CON group, the volume of drainage was significantly reduced in the two treatment groups within 48 h after surgery (*P* < 0.05). compared with the CON group, the postoperative visual analogue scale was significantly lower in groups LIDO and DEX after surgery(*P* < 0.05). Compared with the LIDO group and the CON group, the time to awareness was longer in the DEX group (*P* < 0.05). In the DEX group, bradycardia was noted in 35 patients, while no bradycardia was noted in LIDO group and CON group.

**Conclusion:**

Compared with intravenous infusions of normal saline, both lidocaine and dexmedetomidine had equal effectiveness in attenuating cough and hemodynamic changes during the tracheal extubation period after thyroid surgery, and both of these treatments were able to reduce the volume of postoperative bleeding and provide better analgesic effect after surgery. But intravenous infusions of dexmedetomidine resulted in bradycardia and delayed the time to awareness when compared with lidocaine and normal saline.

**Trial registration:**

ChiCTR1800017482. (Prospective registered). Initial registration date was 01/08/2018.

## Background

It is widely believed that approximately 82.5% of patients experience a cough upon emergence from general anesthesia [1], with causes possibly including the presence of an endotracheal tube, uncleared secretions and anesthetic gas [2]. Cough during tracheal extubation may lead to several complications, such as hypertension, tachycardia, myocardial ischemia and postoperative bleeding [3–5]. Furthermore, postoperative bleeding in thyroid surgery is still significant and is often associated with severe complications including cervical hematoma, reoperation and cardiac arrest [6]. Various strategies aimed at inhibiting cough, including the administration of lidocaine and dexmedetomidine, have been studied [7, 8].

Dexmedetomidine is a potent, alpha-2-selective adrenoceptor agonist, and the most characteristic features include sympatholysis, sedation, analgesia and a lack of respiratory depression [9]. Two studies showed that the administration of single-dose 0.5 mg/kg dexmedetomidine before the end of surgery effectively reduced cough during anesthetic emergence [10, 11]. Additionally, a previous report showed that an intravenous administration of lidocaine can inhibit cough during extubation [12]. Even though both of these treatments have been reported to effectively inhibit cough on the emergence from general anesthesia, but the differences between intravenous lidocaine and dexmedetomidine in inhibiting cough during the tracheal extubation period are unclear.

Therefore, we conducted a study to compare the effects of intravenous infusions of lidocaine and dexmedetomidine in inhibiting cough during the tracheal extubation period after thyroid surgery.

## Methods

### Participants

The Ethics Committee of the Anqing Affiliated Hospital of Anhui Medical University approved the study. This study was registered in the Chinese Clinical Trial Registry (ChiCTR1800017482). Initial registration date was 01/08/2018. Each patient signed an informed consent before surgery. The study took place at the Anqing Affiliated Hospital of Anhui Medical University.

One hundred and-eighty patients were enrolled from August 2018 to November 2018. All of the patients in this study were classified as either American Society of Anesthesiologists (ASA) class I or II, were aged between 18-and 65-years-old from both sexes and were scheduled to undergo thyroid surgery. The exclusion criteria in this study included incidences of asthma, chronic cough, perioperative upper respiratory infection symptoms, a current smoking status, medication involving angiotensin-converting-enzyme inhibitors (ACE-I), bronchodilators or steroid medications, bradycardia or an atrioventricular conduction block, hepatic insufficiency, renal insufficiency, local anesthetic allergy, platelet abnormality, coagulation abnormalities, anticoagulation and a refusal to participate in the study.

Subjects were randomised to the LIDO group, the DEX group and the CON Group with a 1:1:1 allocation using computer-generated random number. Group assignments were kept in sealed envelopes, and only the nurse responsible for preparing the anesthetics was allowed to open the envelope and the assigned drug. The assigned drugs according to group assignments in syringes which has no difference in appearance. The patients, data collectors (anesthesiologist) did not know the drugs used for intravenous administration. All of the patients were NPO since approximately 6 h before surgery.

### Study protocol

All surgeries were performed by three experienced surgeons. All patients received intramuscular hyoscine (0.3 mg) 30 min before the induction of anesthesia. Mean arterial blood pressure (MAP), heart rate (HR), electrocardiogram (ECG) and peripheral pulse oximeter (SPO_2_) values were monitored by using a multiparameter monitor (Philips MIX500, Boeblingen, Germany). In the LIDO group, the patients were given an IV bolus infusion of lidocaine (2%)1.5 mg/kg made to 20 ml with normal saline and 20 ml normal saline respectively, over 10 min before induction of anesthesia, followed by a continuous IV infusion of lidocaine 1.5 mg/kg made up to 20 ml and 20 ml normal saline every hour until 30 min before the end of surgery, respectively. In the DEX group, patients were given IV bolus infusion of dexmedetomidine 0.5 μg/kg made to 20 ml with normal saline and 20 ml normal saline respectively, over 10 min before induction of anesthesia, followed by a continuous IV infusion of dexmedetomidine 0.4 μg/kg made up to 20 ml and 20 ml normal saline every hour until 30 min before the end of surgery, respectively. In the CON group, the patients were given an 20 ml normal saline and 20 ml normal saline respectively, over 10 min before induction of anesthesia, followed by a continuous IV infusion 20 ml normal saline and 20 ml normal saline every hour until 30 min before the end of surgery, respectively. General anesthesia was induced with midazolam (0.05 mg/kg), propofol (2 mg/kg), sufentanil (0.5 μg/kg) and vecuronium (0.1 mg/kg), and anesthesia was maintained with propofol (50–80 μg/kg/min) and remifentanil (0.15–0.2 μg/kg/min). Tracheal intubation was performed after adequate muscle relaxation. All of the patients were ventilated with an Aspire view anesthetic machine (GE Healthcare, Madison, WI, USA). In the three groups, the tidal volume (VT) was maintained at 8 ml/kg, the respiratory rate (RR) was fixed at 12 breaths/min, the inspiratory to expiratory time ratio (I: E) was 1:2 and the inspired oxygen fraction (FiO_2_) was 0.5 (balanced with air) throughout the anesthesia period. To maintain a controlled ventilation, vecuronium was intermittently used for muscle relaxation. The depth of anesthesia was maintained with an infusion rate of propofol and remifentanil, according to the Bispectral Index values (BIS) and the hemodynamic parameters within 20% of the baseline. To prevent the occurrence of intraoperative awareness, the BIS values were kept between 45 and 60 in the three groups during surgery. Neuromuscular blocks were reversed with atropine (0.5 mg) and neostigmine (1 mg) before the tracheal extubation. Experienced surgeons preserved the anatomical integrity of motor nerves by visual identification and exposure both of the external branch of the superior laryngeal nerve and the recurrent laryngeal nerve, and the recurrent laryngeal nerve was prevented injury by intraoperative neuromonitoring during thyroid surgery. After the tracheal extubation, all of the patients were transferred to the post anesthesia care unit (PACU).

### Data collection

Demographic and clinical characteristics, including age, height, weight, ASA grade, gender, PLT (platelet), APTT (activated partial thromboplastin time), PT (prothrombin time), TT (thrombin time), Fib (fibrinogen) were recorded. Intraoperative fluid input, intraoperative blood loss and intraoperative urine output were recorded. The incidence and severity of cough within 5 min during the extubation was recorded: 0 = no cough, 1 = minimal (single) cough, 2 = moderate (≤5 s) cough and 3 = severe (> 5 s) cough (bucking) [13]. The MAP and HR were measured and recorded before induction, during tracheal extubation and 5 min after tracheal extubation. The time to awareness, the postoperative length of hospital and any adverse events including local anesthetic toxicity, supraventricular or ventricular arrhythmias, bradycardia (HR < 60beat/min), hypotension (systolic blood pressure < 90 mmHg), need for vasopressors and prolonged respiratory support were recorded. Volume of drainage within the first and second 24 h after surgery, cervical hematoma, need for surgical revision, need for transfusion and time to removal of drainage were recorded. Patients were assessed in surgical ward for pain intensity using a 10 cm visual analogue scale (VAS: 0 = no pain, 10 = the most imaginable pain).

### Statistical analysis

Calculation of sample size was based on the incidence of cough. In the pilot study, the two treatments infusion reduced the incidence of cough by 35%, and incidence of cough in the CON group was 62% and an α of 0.05, 55 patients would be required in each group (assuming a power of 0.80). Anticipating a study drop-out rate of 10%, we included 60 patients per group.

Data analysis was performed by using SPSS for Windows V.16.0 (SPSS Inc., Chicago, IL). Data were expressed as numbers, percentages or means±standard deviations. The quantitative variables were performed by using a one-way ANOVA with post hoc analysis. Repeated measurements were analysed using linear mixed model with a Bonferroni correction. Intergroup differences of the parameters at each time point were determined by using a one-way ANOVA with a post hoc analysis. The qualitative data were presented as numbers/percentages, and analysed by using a χ^2^ test. *P* values of less than 0.05 were considered to be statistically significant.

## Results

**Fig. 1:**
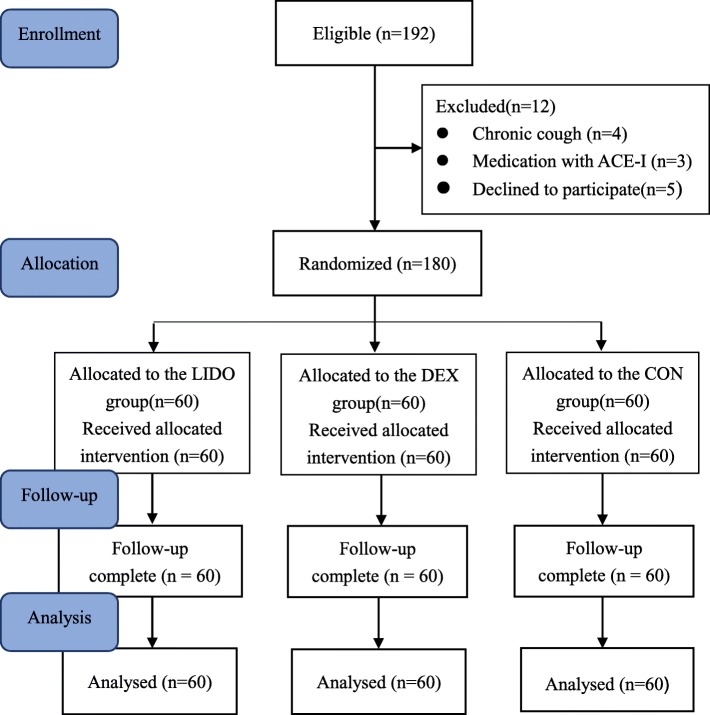
CONSORT flow diagram for the study

A total of 192 patients were assessed for eligibility for the study, and 180 subjects were enrolled in the study (Fig. 1). Twelve patients were excluded (reasons for exclusion are listed in Fig. 1). There were no significant differences among the three groups with respect to age, weight, height, ASA class, sex, APTT, PT, TT, Fib, duration of anesthesia, duration of surgery, intraoperative fluid input, intraoperative blood loss and intraoperative urine output. (Table 1). The incidences of cough were significantly lower in the LIDO group (28.3%) and the DEX group (31.7%) than in the CON group (66.7%) (*P* = 0.000). Additionally, both moderate and severe cough were significantly lower in the LIDO group (13.3%) and the DEX group (13.4%) than in the CON group (43.4%) *(P* < 0.05). There were no differences in the incidence and severity of cough between the two treatment groups (Table 2). Compared with the LIDO group and the DEX group, both MAP and HR were significantly increased in the CON group during tracheal extubation and 5 min after tracheal extubation (*P* < 0.05). There were no differences in MAP or HR between the two treatment groups (Table 3). The time to awareness in the DEX group were longer than that in the LIDO group and the CON group, while the postoperative length of hospital stays in the CON group than that in the LIDO group and the DEX group. No adverse effects including local anesthetic toxicity, supraventricular or ventricular arrhythmias, hypotension, need for vasopressors and prolonged respiratory support were observed in the study. In the DEX group, bradycardia (HR < 60 beat/min) was noted in 35 patients (58.3%) without hypotension, and one patient’s HR was reduced by 40 beat/min, and that was treated with atropine 0.5 mg iv. No bradycardia was noted in LIDO group and CON group. No patients required prolonged respiratory support after the tracheal extubation in the three groups. Compared with the CON group, the volume of drainage was significantly reduced in the LIDO group and the DEX group within the first and second 24 h after surgery (*P* < 0.05), and there was no difference in the volume of drainage between the two treatment groups (Table 4). All drainages in the LIDO group and DEX group were removed within 48 h after surgery, while 60% (36 cases) drainages in the CON group were removed. There was a 1.7% incidence of cervical hematoma and need for surgical revision without transfusion after surgery in the CON group. Compared with the LIDO group and the CON group, the time to awareness was longer in the DEX group(*P* < 0.01). Compared with the LIDO group and the DEX group, the postoperative length of hospital stay was longer in the CON group(*P* < 0.01) (Table 5). The VAS scores in the LIDO group and the DEX group were lower than these in the CON group in any time point after surgery(*P* < 0.01) (Table 6).

**Table 1:** Demographic and clinical characteristics

Variables	LIDO group	DEX group	CON group	*P* value
	(*n* = 60)	(*n* = 60)	(*n* = 60)	
Age (yr)	48.4 ± 8.8	47.6 ± 7.8	49.3 ± 7.2	0.661
Weight (kg)	58.8 ± 6.9	57.6 ± 5.7	60.1 ± 6.4	0.320
Height (cm)	158.6 ± 5.1	157.7 ± 4.5	158.9 ± 6.1	0.815
ASA class (I/II)	55/5	58/2	57/3	0.477
Gender, Female/Male	35/25	37/23	34/26	0.933
PLT(10 × 10^9^/L)	197.3 ± 39.9	198.5.6 ± 34.2	181.044.8	0.412
PT(s)	10.6 ± 0.8	10.4 ± 0.8	10.8 ± 0.6	0.280
APTT(s)	27.5 ± 2.6	26.3 ± 4.2	27.4 ± 2.5	0.524
Fib(g/L)	2.4 ± 0.7	2.2 ± 0.4	2.1 ± 0.3	0.143
Duration of anesthesia (min)	82.1 ± 19.4	92.2 ± 25.5	81.8 ± 20.4	0.242
Duration of surgery (min)	99.4 ± 20.7	111.4 ± 30.8	104.0 ± 24.1	0.333
Intraoperative fluid input (mL)	691.0 ± 155.9	638.0 ± 151.3	725..0 ± 170.6	0.229
Intraoperative blood loss (mL)	59.9 ± 12.2	61.9 ± 11.3	65.7 ± 12.3	0.368
Intraoperative urine output (mL)	447.5 ± 90.1	428.9 ± 98.5	423.8 ± 80.5	0.682

Categorical variables were expressed as the mean ± standard deviation (SD) or numbers. LIDO group, iv. lidocaine; DEX group, iv. dexmedetomidine; CON group, iv. equal volume normal saline

**Table 2:** Incidence and grade of cough

Variables	LIDO group	DEX group	CON group	*P* value
	(*n* = 60)	(*n* = 60)	(*n* = 60)	
Incidence of cough, n (%)	17 (28.3) ^*^	19 (31.7) ^*^	40 (66.7)	0.000
Grade 0	43 (71.7) ^*^	41 (68.3) ^*^	20 (33.3)	0.000
Grade 1	9 (15.0)	11 (18.3)	14 (23.3)	0.502
Grade 2	6 (10.0) ^**^	5 (8.4) ^**^	16 (26.7)	0.008
Grade 3	2 (3.3) ^**^	3 (5.0) ^**^	10 (16.7)	0.016

Categorical variables were expressed as numbers (proportions). LIDO group, iv. lidocaine; DEX group, iv. dexmedetomidine; CON group, iv. equal volume normal saline. The severity of cough was evaluated during the recovery period from the time of awareness to 5 min after extubation: 0 = no cough, 1 = minimal (single) cough, 2 = moderate (≤5 s) cough and 3 = severe (> 5 s) cough (bucking)

^*^P = 0.000 vs the CON group; ^**^P < 0.05 vs the CON group

**Table 3:** MAP and HR change

Variables	LIDO group	DEX group	CON group	*P* value
	(*n* = 60)	(*n* = 60)	(*n* = 60)	
MAP (mmHg)				
Before induction	86.9 ± 12.6	83.6 ± 10.4	87.6 ± 13.4	0.167
During tracheal extubation	84.8 ± 14.4	88.2 ± 14.5	101.4 ± 13.3^*^	0.000
5 min after tracheal extubation	91.7 ± 16.5	90.8 ± 13.1	104.7 ± 15.7^*^	0.000
HR (beat/min)				
Before induction	79.8 ± 10.4	83.5 ± 13.4	83.6 ± 15.3	0.198
During tracheal extubation	80.7 ± 12.4	79.4 ± 8.1	95.3 ± 13.6^*^	0.000
5 min after tracheal extubation	86.6 ± 13.8	85.2 ± 11.6	101.1 ± 15.6^*^	0.000

Categorical variables were presented as the mean ± standard deviation for all of the patients, with 60 cases in each group. LIDO group, iv. lidocaine; DEX group, iv. dexmedetomidine; CON group, iv. equal volume normal saline. MAP, mean arterial pressure; HR, heart rate. Compared with the LIDO group and the DEX group, both MAP and HR were significantly increased in the CON group during tracheal extubation and 5 min after tracheal extubation (^*^P = 0.000)

**Table 4:** Volume of drainage within 48 h after surgery

Variables	LIDO group	DEX group	CON group	*P* value
	(*n* = 60)	(*n* = 60)	(*n* = 60)	
Volume of drainage (mL)				
within the first 24 h after surgery	68.3 ± 10.5^*^	71.0 ± 13.7^*^	108.1 ± 18.9	0.000
within the second 24 h after surgery	23.9 ± 7.8^*^	24.2 ± 6.4^*^	51.0 ± 29.6	0.000

Categorical variables were presented as the mean ± standard deviation for all of the patients. LIDO group, iv. lidocaine; DEX group, iv. dexmedetomidine; CON group, iv. equal volume normal saline, with 60 cases in each group. Compared with the CON group, the volume of drainage was significantly reduced in the LIDO group and the DEX group (^*^P = 0.000)

**Table 5:** Recovery profile after the surgery

Variables	LIDO group	DEX group	CON group	*P* value
	(*n* = 60)	(*n* = 60)	(*n* = 60)	
Time to awareness (min)	10.2 ± 1.7	19.1 ± 2.6^*^	9.3 ± 2.2	0.000
Postoperative length of hospital stay (d)	3.4 ± 0.9	3.6 ± 0.9	5.0 ± 1.5^*^	0.001

Categorical variables were presented as the mean ± standard deviation for all of the patients, with 60 cases in each group. LIDO group, iv. lidocaine; DEX group, iv. dexmedetomidine; CON group, iv. equal volume normal saline. Time to awareness = time from discontinuation of propofol and remifentanil to spontaneous eye opening by light stimulation. Compared with the LIDO group and the CON group, the time to awareness was longer in the DEX group (^*^P = 0.000). Compared with the LIDO group and the DEX group, the postoperative length of hospital stay was longer in the CON group (^*^P < 0.01)

**Table 6:** VAS pain scores at any point time after the surgery

Variables	LIDO group	DEX group	CON group	*P* value
	(*n* = 60)	(*n* = 60)	(*n* = 60)	
VAS scores				
At 2 h	2.1 ± 0.4	1.9 ± 0.3	3.6 ± 0.7^*^	0.000
At 4 h	2.4 ± 0.5	2.1 ± 0.6	3.9 ± 0.8^*^	0.000
At 8 h	2.6 ± 0.3	2.3 ± 0.5	5.4 ± 0.6^*^	0.000
At 12 h	2.3 ± 0.5	2.2 ± 0.6	5.7 ± 0.8^*^	0.000
At 24 h	2.0 ± 0.3	2.0 ± 0.5	4.1 ± 0.5^*^	0.000

Categorical variables were presented as the mean ± standard deviation for all of the patients, with 60 cases in each group. LIDO group, iv. lidocaine; DEX group, iv. dexmedetomidine; CON group, iv. equal volume normal saline. Compared with the LIDO group and the DEX group, VAS pain scores were higher in the CON group (^*^P < 0.01)

## Discussion

This study demonstrated that intravenous infusions of lidocaine and dexmedetomidine were effective in attenuating cough and hemodynamic changes during the tracheal extubation period in patients undergoing thyroid surgery without side effects such as anesthetic toxicity, supraventricular or ventricular arrhythmias, intraoperative hypotension, and prolonged respiratory support. Additionally, both of these treatments were able to reduce the volume of postoperative bleeding and provide satisfactory analgesic effect after surgery. But intravenous infusions of dexmedetomidine resulted in bradycardia and delayed time to awareness.

Lidocaine has several beneficial effects, such as analgesia, anti-hyperalgesia and anti-inflammation [14, 15]. Moreover, lidocaine can depress spike activity, amplitude and conduction time in both myelinated A and unmyelinated C nerve fibers [16]. Several studies have shown that lidocaine can reduce the incidence and severity of cough during anesthetic emergence through different methods, including intracuff, tube lubrication, intratracheal instillation and intravenous bolus infusions before an induction [17–20]. Shabnum et al. [12]. found that both IV and intratracheal lidocaine are effective in the attenuation of cough. In our study, the incidence and severity of cough was 28.3% in the LIDO group, and the rate of cough was significantly lower than the rate in a previous study (72.1%) [8]. We speculated that the methods of intravenous infusion of lidocaine might contribute to the difference. The effective serum concentration of lidocaine for the attenuation of cough is between 2.3 μg/ml and 3.0 μg/ml [21], and it is difficult to achieve this concentration in a timely manner via bolus infusion administration; however, the target concentration can likely be obtained by extending the intravenous infusion time. The present study demonstrated that the intravenous infusion of lidocaine could effectively suppress cough during the tracheal extubation period.

Several studies have shown that dexmedetomidine can effectively reduce cough during anesthetic emergence [8, 10], but the exact mechanism is unclear. A previous study has shown that a peripheral alpha-2 receptor may be involved in cough inhibition [22]. In addition, a previous study showed that the sedative characteristics of dexmedetomidine can suppress the sensitivity of tracheal stimulation, which then results in cough inhibition [23]. However, several studies have shown that a dexmedetomidine infusion, at a rate of 0.4 μg/kg/h during the operation period, did not inhibit cough [24, 25]. Park et al. [23]. compared the effect of a single dose of 0.5 μg/kg dexmedetomidine with remifentanil by the use of a target-controlled infusion in reducing cough during anesthetic emergence. The results of this study showed that the effect of dexmedetomidine was lower than that of remifentanil. In addition to the administration of a loading dose of infusion before the induction of anesthesia, a continuous infusion administration was also given until 30 min before the end of surgery in the DEX group, so the incidence of cough decreased by 35%, which thus contributed to the sedative effect of dexmedetomidine, but the sedative effect could delay the time to awareness.

The thyroid gland has both a rich vascular supply and high blood perfusion, bleeding after thyroid surgery occurs more often than after other surgical procedures. Postoperative bleeding usually occurs within 12 h, and especially occurs within 6 h after surgery [26], And coughing may increase the risk of postoperative bleeding. Although suction drain was commonly used in thyroidectomy, but drains’ value in removing blood, not value in developed bleeding. Furthermore, bleeding after thyroid surgery is still significant and is often associated with severe complications including cervical hematoma, reoperation and cardiac arrest [6]. In the CON group, there was a 1.7% incidence of cervical hematoma and need for surgical revision. Reductions of postoperative bleeding and potential consequences contributed to patients’ recovery who underwent thyroid surgery [27]. In our study, the volume of drainage within 48 h after surgery was lower in the two treatment groups than that in the CON group, as a result that the time to removal of drainage and the postoperative length of hospital stay in the CON group were longer than these in two treatment groups.

The stimulation of the respiratory tract by an endotracheal tube during an endotracheal extubation causes transient sympathetic activity, which can lead to hypertension and tachycardia [28]. Various attempts have been made to attenuate the pressor response via intravenous administrations of lidocaine and dexmedetomidine. A previous study reported that intravenous lidocaine can blunt increases in HR and MAP during the tracheal extubation [29]. Luthra et al. [30]. demonstrated that intravenous dexmedetomidine can alleviate stress responses to tracheal extubation. In our study, both MAP and HR were decreased in the LIDO group and the DEX group during extubation and 5 min after extubation, compared to the CON group. But because of the sympatholysis, intraoperative bradycardia was noted in 35 patients, and one patient’s HR was reduced by 40 beat/min during intravenous infusion of dexmedetomidine in the DEX group.

Both intravenous infusions of lidocaine and dexmedetomidine could target smooth emergence from general anesthesia through attenuating cough and hemodynamic changes, and provide satisfactory analgesic effect after thyroid surgery. The VAS scores in the LIDO group and the DEX group were lower than these in the CON group after surgery. These findings may be explained by the analgesic properties of both lidocaine and dexmedetomidine.

There were several limitations in this study. First, the consumptions of anesthetic agents were not evaluated; however, both lidocaine and dexmedetomidine have analgesic properties. Second, this study was a single-center clinical study, and the conclusions still need to be further supported by large sample and multicenter studies.

## Conclusions

This study was demonstrated that both intravenous infusions of lidocaine and dexmedetomidine had equal effectiveness in attenuating cough and hemodynamic changes during the tracheal extubation period after thyroid surgery, and both of these treatments were able to reduce the volume of postoperative bleeding and provide satisfactory analgesic effect after surgery. But intravenous infusions of dexmedetomidine resulted in bradycardia and delayed the time to awareness.

## Abbreviations

ACE-I: Angiotensin-converting-enzyme inhibitors
APTT: Activated partial thromboplastin time
ASA: American Society of Anesthesiologists
BIS: Bispectral index
CON: Control
DEX: Dexmedetomidine
ECG: Electrocardiogram
Fib: Fibrinogen
FiO_2_: Inspired oxygen fraction
HR: Heart rate
LIDO: Lidocaine
MAP: Mean arterial blood pressure
PACU: post anesthesia care unit
PLT: Platelet
PT: Prothrombin time
RR: Respiratory rate
SPO_2_: Peripheral pulse oximeter values
TT: Thrombin time
VAS: Visual analogue scale
VT: Tidal volume
